# Dengue and Dengue Hemorrhagic Fever, Brazil, 1981–2002

**DOI:** 10.3201/eid1101.031091

**Published:** 2005-01

**Authors:** João Bosco Siqueira, Celina Maria Turchi Martelli, Giovanini Evelim Coelho, Ana Cristina da Rocha Simplício, Douglas L. Hatch

**Affiliations:** * Ministry of Health, Brasília, Brazil; † Federal University of Goiás, Goiânia, Brazil; ‡ Centers for Disease Control and Prevention, Atlanta, Georgia, USA

**Keywords:** Dengue, Dengue hemorrhagic fever, Surveillance, Brazil, research

## Abstract

In the last 5 years, Brazil has accounted for ≈70% of reported dengue fever cases in the Americas. We analyzed trends of dengue and dengue hemorrhagic fever (DHF) from the early 1980s to 2002 by using surveillance data from the Brazilian Ministry of Health. Two distinct epidemiologic patterns for dengue were observed: localized epidemics (1986–1993), and endemic and epidemic virus circulation countrywide (1994–2002). Currently, serotypes 1, 2, and 3 cocirculate in 22 of 27 states. Dengue and DHF affected mainly adults; however, an increase in occurrence of DHF among children has been recently detected in northern Brazil, which suggests a shift in the occurrence of severe disease to younger age groups. In 2002, hospitalizations increased, which points out the change in disease severity compared to that seen in the 1990s. We describe the epidemiology of dengue in Brazil, characterizing the changing patterns of it and DHF during the last 20 years.

## Abstract 2

Brazil has experienced an increase in dengue disease severity in the past 5 years.

Brazil accounted for nearly 70% of the 3,141,850 reported cases of dengue fever in the American regions in the last 5 years (*1*). Dengue fever and a severe manifestation of the illness known as dengue hemorrhagic fever/dengue shock syndrome (DHF/DSS) are increasingly important health, environmental, and economic concerns in the Americas (*2**,**3*). Dengue/DHF is a febrile illness caused by a flavivirus with four known serotypes (dengue virus [DENV]1, 2, 3, 4), and infection is mainly transmitted by the mosquito *Aedes aegypti* (*4*). Currently, no vaccine is available for dengue fever, and disease control and prevention have mainly focused on vector control activities and surveillance that incorporates community participation (*5**,**6*). Despite vector control programs and heightened public awareness, outbreaks have occurred in several highly urbanized areas in Central and South America (*7**–**9*).

The reemergence of dengue in Latin America occurred during the 1960s with epidemics in the Caribbean and Venezuela and in the 1970s in Colombia (*3*). Brazil remained free of *A. aegypti* until 1976 as a result of the successful eradication program to prevent urban yellow fever coordinated by the Pan American Health Organization (PAHO) in previous decades (*10**,**11*). The subsequent reinfestation of *A. aegypti* into urban areas of Brazil and the introduction of DENV in 1986 led to resurgence of dengue fever outbreaks and an increased risk for urbanization of yellow fever in the country (*12*).

The Brazilian system for dengue/DHF reporting relies on passive surveillance, with laboratory diagnosis for case ascertainment and identification of circulating serotypes. A parallel system for entomologic surveillance exists to monitor virus and vector dispersion. Analyses of data reported to the state and national level are used to evaluate the impact of disease, time trends, and geographic distribution, with the objective of supporting and improving public health interventions (*13*). In a previous report, we analyzed the dengue situation, focusing on the main policies regarding prevention and control strategies adopted in Brazil (*8*). Here, on the basis of national surveillance data, we analyze the trends of dengue/DHF from the early 1980s to 2002 and contrast the changes in the epidemiologic pattern of disease for regions in Brazil.

## Methods

Brazil is the largest and most populated country in Latin America, covering >8 million km^2^ with an estimated 2002 population of 174,632,932 inhabitants. High population density areas and cities (up to 12,901 inhabitants/km^2^) are located mainly on the Atlantic Coast. Most of Brazil has a tropical climate; in the southern region, the climate is subtropical. The rainy season is observed in the first several months of the year, and the average temperature is >20°C (*14*).

### Sources of Data

Dengue/DHF is a reportable disease in Brazil, and the ministry of health has implemented a surveillance system since the first epidemic in the early 1980s. We reviewed available data compiled from 1986 to 2003 by the official surveillance system. We also used the available computerized data from the Hospitalization Information System of the Unified Health System (SIH-SUS), which covers ≈70%–80% of overall hospitalizations in the country (*15**,**16*). This system permits recovery of information on hospital admissions according to the International Disease Classification system (ICD versions 9 and 10). For this analysis, we selected hospitalizations due to dengue fever and DHF stratified by state from 1990 to 2003.

### Case Definition and Surveillance Forms

During the study period, case definitions used for suspected, probable, and confirmed dengue/DHF adhered to PAHO guidelines (*17*). Information on persons with suspected dengue/DHF is routinely reported by using standardized forms completed by clinicians or health staff and subsequently sent to local health surveillance officials for data checking. This dengue/DHF case investigation form includes information on basic demographic data, dates of symptom onset and sample collection, case classification (dengue fever, DHF, DSS, or discarded case), and outcome. Individual data are locally entered into the electronic information system and subsequently transmitted to state and national levels.

### Laboratory Confirmation and Entomologic Surveillance

Laboratory confirmation was based on the following: 1) serologic tests (immunoglobulin M antibody-capture enzyme-linked immunosorbent assay) performed on serum samples collected >6 days after the onset of symptoms (*18*); or 2) virus isolation in C6/36 mosquito cell culture from blood samples collected <6 days after symptom onset (*19*). Immunohistochemical studies with formalin-fixed tissues are performed on selected patients who died (*20*).

Initially, three public health laboratories were responsible for DENV laboratory confirmation: Evandro Chagas Institute (located in Belém, Pará state), which is the ministry of health reference laboratory; Oswaldo Cruz Foundation (Rio de Janeiro); and Adolfo Lutz Institute (São Paulo). During the 1990s, the Public Health Laboratory Network was set up for the dengue-endemic states to respond to increasing requests for laboratory confirmation. By 2001, a total of 27 public laboratories at state level were in charge of serologic tests and quality control. Information on circulating serotypes was obtained from viral isolation by the 3 national reference laboratories during the 1980s and subsequently from 13 public health laboratories at the state level. Laboratory results are routinely linked to individual data. Classification of DHF required laboratory confirmation throughout the study period. Approximately 30% of dengue fever cases are also laboratory-confirmed. During epidemics, dengue cases were mostly classified by clinical and epidemiologic criteria because of limits in laboratory capacity.

The number of municipalities documented with *A. aegypti* infestations and information on vector dispersion were abstracted from data compiled by the national vector information system for yellow fever and dengue.

### Data Analysis

A descriptive analysis of dengue incidence was performed by region (using residence of reported cases) from 1986 to 2002. Incidence and hospitalization rates by age group were calculated by using census population data as denominators. The ratio of incident versus hospitalized cases was also calculated. Exploratory data analysis of the age group of reported patients focused on data from 1998 to 2002, since standardized data in electronic format for these variables were available for this period.

## Results

**Figure 1:**
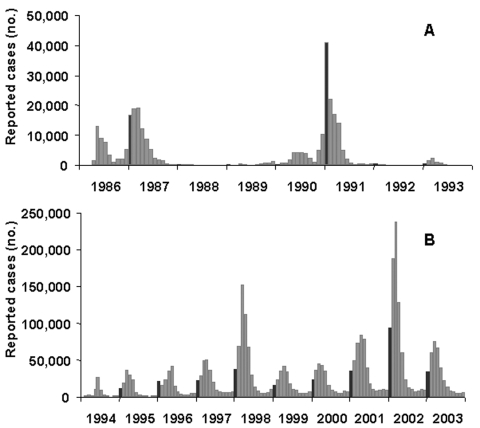
Number of dengue fever cases reported per month, Brazil. A) 1986–1993, B) 1994–2002. Dark bars represent January.

**Figure 2:**
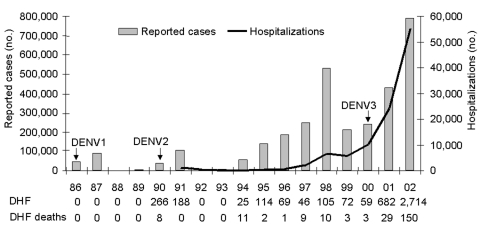
Number of reported cases and hospitalizations due to dengue and dengue hemorrhagic fever (DHF), Brazil, 1986–2002.

The epidemiologic pattern of dengue fever in Brazil during the last 20 years can be divided into 2 distinct periods: 1) epidemic waves in localized areas (1986–1993) and 2) epidemic and endemic virus circulation countrywide (1994–2002) (Figure 1 and Table 1). The main dengue-related events occurring in these 2 periods are summarized in Table 1. In 1981, the first laboratory-confirmed cases of dengue (DENV1 and DENV4) occurred in an isolated area in the northwest Amazon region (Roraima State). After a 5-year interval without confirmed dengue fever, an epidemic due to DENV1 occurred in Rio de Janeiro State and was followed by several epidemics in highly populated cities in the southeast and northeast regions of Brazil. The number of dengue fever cases peaked at ≈100,000 in 1987 and 1991, probably because different serotypes (DENV1 and DENV2, respectively; Figure 2) were introduced. From 1986 to 1993, a total of 76.6% of the 294,419 reported dengue cases occurred in the rainy season from December to May, showing a marked seasonal pattern (Figure 1B). A cyclical pattern of 2-year intervals between large outbreaks was observed, which suggested low viral activity in the dry season (June to November).

**Table 1:** Chronology of major dengue-related events in Brazil, 1981–2002

Period/y	Event
1981–1993: Epidemics waves in localized areas	
1981	Restricted outbreak (DENV1* and DENV4†) in Northwest Brazil (Roraima State)
1986	Introduction of DENV1 (Rio de Janeiro State)
1990	Introduction of DENV2‡ (Rio de Janeiro State) and first confirmed cases of DHF
1994–2002: Epidemic and endemic virus circulation countrywide	
1994–1999	Dispersion of *Aedes aegypti* nationwide
1998	Widespread outbreaks in 16 states (>534,000 reported cases)
2000	Introduction of DENV3§ (Rio de Janeiro State)
2002	Large outbreaks in 19 states (>794,000 reported cases) Deaths due to dengue hemorrhagic fever exceed deaths from malaria

*Dengue virus serotype 1. †Dengue virus serotype 4. ‡Dengue virus serotype 2. §Dengue virus serotype 3.

In the second period (1994–2002), a total of 2,826,948 dengue cases were reported, indicating an upward trend in the incidence from 37 to 454 per 100,000 inhabitants. Although large outbreaks were observed in the rainy season, 482,163 cases were reported in the nonrainy season, which demonstrated increased dengue virus activity during the entire year. The bulk of incident cases were generated from metropolitan areas, although several outbreaks occurred in smaller urban settings in 25 out of 27 states. Two unprecedented epidemics occurred in 1998 and 2002, with 528,388 and 794,219 dengue fever cases reported, respectively. During this second period, vector surveillance to monitor *A. aegypti* infestation was extended to most of the country, covering 69.7% (n = 3,878) of the municipalities in 1996 and 89.6% (n = 4,985) in 2002. According to this vector information system, the number of infested municipalities ranged from 1,726 (44.5%) in 1996 to 2,905 (58.3%) in 2002. To date, Santa Catarina and Rio Grande do Sul States, located in southernmost Brazil, remain free of autochthonous dengue transmission; they report only imported cases.

**Figure 3:**
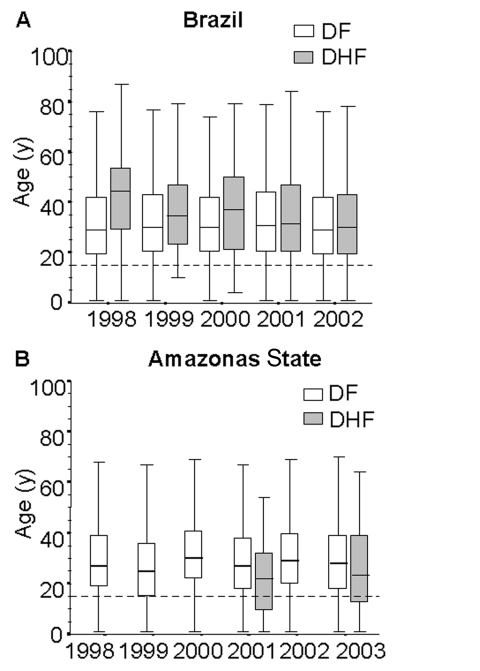
Age distribution of dengue fever (DF) and dengue hemorrhagic fever (DHF) for Brazil and Amazonas State, 1998–2002. Boxes encompass 25th and 75th percentiles. Black lines within boxes, medians. Outliers not shown. Dashed line, 15 years old.

The overall age distribution among reported cases during the last 5 years is presented in Figure 3. Approximately 50% of all reported dengue cases occurred in adults 20–40 years of age. During this period, dengue incidence was consistently higher in adults, reaching up to 432.7/100,000 population in the 30- to 49-year-old age group in 2002 (Table 2). The male:female ratio was constant at ≈1.1:1 during this 5-year period. Sex and age group distribution were very similar when the data for the 5 regions of the country were stratified (data not shown).

**Table 2:** Dengue incidence and hospitalization (per 100,000 population) by age group, Brazil, 1998–2002

Age (y)	1998		1999		2000		2001		2002	
	Incidence	Hosp*	Incidence	Hosp	Incidence	Hosp	Incidence	Hosp	Incidence	Hosp
0–4	39.6	1.1	27.2	0.5	29.6	1.1	68.8	2.9	121.9	10.3
5–14	68.2	2.1	45.4	1.6	51.8	3.0	131.7	7.0	213.7	19.6
15–29	145.6	4.5	102.7	4.1	111.3	7.5	263.5	16.6	410.3	36.6
30–49	156.3	4.7	120.3	4.4	128.0	7.2	305.7	16.7	432.7	36.1
>50	120.0	6.5	92.0	5.7	88.3	8.4	237.3	20.7	323.6	43.7
Total	117.2	4.0	85.7	3.5	92.3	6.0	225.3	14.1	335.3	31.6

*Hospitalization rates.

The first DHF cases were confirmed in 1990, after DENV2 was introduced into Brazil. During the decade that followed, 893 confirmed DHF cases with 44 deaths (rate of 4.9%) occurred; 75% of these deaths occurred in Rio de Janeiro State. During 2001–2002, a striking increase in the number of DHF cases was detected, with incidence rates of 2.9/100,000 population (n = 682) in 2001 and 12.9/100,000 population (n = 2,714) in 2002 (Figure 2). DHF cases increased 45-fold from 2000 to 2002, compared to a 3.3-fold increase in dengue fever cases during the same period. In the epidemic year of 2002, the overall ratio of dengue fever to DHF cases was 292.6; in Rio de Janeiro State, the most affected area, this ratio was 134.8. From 1998 to 2002, the case-fatality rate was 5.4% (195/3,632). During this period, the sex distribution of DHF was similar, and cases occurred mainly in adults (median age 33 years) (Figure 3A). Recently, a different pattern in the age distribution of DHF was observed in Amazonas State, where unlike the national level, a higher proportion of DHF cases occurred among children. In Amazonas State, 30.9% (17/55) and 28.8% (15/52) of DHF cases occurred among children <15 years of age in 2001 and 2003, respectively (Figure 3B).

**Figure 4:**
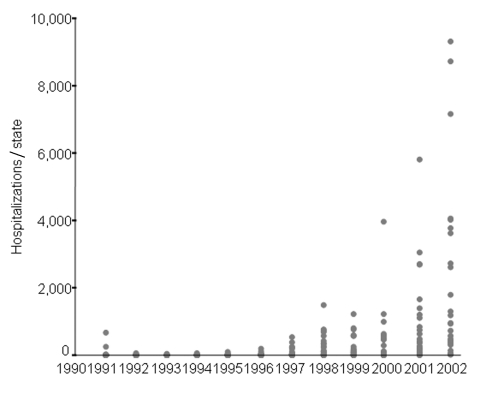
Number of dengue fever–related hospitalizations per state by year, Brazil, 1990–2003. Each dot represents the number of hospitalizations due to dengue/dengue hemorrhagic fever for 1 state by year. Source: Hospitalization Information System–Unified Health System (SIH-SUS).

Countrywide, an upward trend in hospitalized dengue cases has been apparent since 1994, peaking with >54,000 hospitalizations in 2002 (Figures 2 and 4). Hospitalization rates were consistently higher among adults from 1998 to 2002. Comparing the 2 largest epidemic years (1998 and 2002), hospitalization rates in all age groups increased ≈8-fold (Table 2). The ratio of reported to hospitalized cases decreased from 29.4 in 1998 to 10.6 in 2002, which represents 1 hospitalization for every 10 reported dengue cases. Despite this increased proportion of hospitalized cases, >90% of the total reported cases were considered mild or moderate cases by the health system.

In Brazil, the chronology of the appearance of DENV serotypes occurred in the following sequence: DENV1 in 1986, DENV2 in 1990, and DENV3 in 2000 (Figure 2). The simultaneous circulation of these three serotypes was first detected in multiple states during the last 2 years. Molecular epidemiology of DENV in Brazil has identified the Caribbean, Jamaican, and Sri Lankan genotypes for the DENV1, DENV2, and DENV3 viruses, respectively (*21*).

## Discussion

Analysis of 20 years of dengue/DHF surveillance data showed 2 distinct periods: an initial phase from 1986 to 1993, characterized by localized, sporadic epidemic waves in urban centers, and a nationwide endemic and epidemic pattern from 1994 to 2002. In this latter period, several epidemics of dengue progressed towards hyperendemicity in multiple urban centers. Since 1999, dramatic increases in both the incidence and hospitalizations due to dengue fever and DHF have occurred, indicating a likely increase in disease severity in recent years. Changes in surveillance coverage might explain part of this increase, but this explanation seems unlikely since no major changes were made in surveillance definitions. The current intense virus transmission pattern can be explained by the number of municipalities infested with *A. aegypti* mosquitoes, the mobility of the population, and the introduction and cocirculation of 3 different virus serotypes (DENV1, 2, and 3).

The epidemiology of dengue in Brazil presents some unusual features, characterized by dengue/DHF affecting mainly adults, with a predominance of milder infection in persons treated at outpatient clinics. In contrast, severe forms of the disease among children requiring hospitalization have been described in DHF epidemics in South and Central American countries (*3**,**22**,**23*). This endemic pattern has also been reported from Southeast Asia in recent decades (*24**,**25*). Cuban children with secondary infection by DENV2 showed a higher frequency of DHF and DHF-related deaths during the epidemic of 1981, which had been preceded by a DENV1 outbreak 4 years earlier (*26*). Apparently, similar events in Brazil, which also experienced the sequential introduction of DENV1 (during 1986) followed by DENV2 4 years later, led to a different outcome: most reported dengue/DHF occurred in adults. These findings may be explained by the distinct DENV2 strains circulating in Cuba (New Guinea) and Brazil (Jamaica) (*21**,**27**,**28*). Another possibility is the underdiagnosis of DHF in children in Brazil, although it seems unlikely that severe clinical manifestations would not likely be misdiagnosed or fail to draw the attention of public health authorities.

During the 20-year period examined, dengue/DHF in Brazil most commonly affected the adult population, even with the observed increase of dengue/DHF cases. Despite these findings, current data underscore a different pattern in the Amazon region, where an increased proportion of severe cases occurs among children. This finding represents a warning to pediatric practitioners and health officials. In the future, if the current intense DENV circulation persists, Brazil could resemble Southeast Asia, with DHF occurring mainly in younger age groups.

In 2002, the absolute number of deaths due to DHF (N = 150) exceeded malaria deaths for the first time in Brazil, demonstrating that malaria is not the only major endemic vector-borne disease in this tropical region. During that year, the largest epidemic yet recorded, including 250,000 dengue cases in the metropolitan area of Rio de Janeiro, caused major public health and political concern (*7**,**8*). Hemorrhagic fever and dengue-related deaths also clustered in this city. These findings probably reflect the recent introduction and predominance of DENV3, which suggests a possible role of this serotype in disease severity and the potential for additional DHF epidemics in the future (*29*). Another possible explanation for the increase in DHF is the association between secondary infection with disease severity (*10*); however, routine surveillance data only indicate patients’ immune status. In addition, some highly urbanized areas have substantial proportions of the population living in crowded, impoverished areas with poor sanitation (*30*). These complex urban settings are present in many regions in Brazil, resulting in a major challenge for vector control activities (*8*).

Several seroepidemiologic studies in Brazil have shown that up to 70% of urban populations had previous dengue infection, outnumbering reported cases. These results suggest that seroprevalence increases with age and that subclinical outcomes are a common feature of DENV exposure (*31**,**32*). Access to health services is considered nearly universal in Brazil, particularly in urban settings; therefore, dengue/DHF reports should be representative of the disease in the population. Incidence and hospitalization rates by age group showed similar patterns when surveillance system and hospitalization database were analyzed. The consistent patterns of age and sex distribution for all regions during epidemic and nonepidemic periods also suggest reliability of these surveillance data.

The high case-fatality rate for DHF observed in the last 5 years may be due to difficulties in classifying case severity according to the standard World Health Organization/PAHO dengue case definition (*33*). The Brazilian Ministry of Health has been strongly committed to improving surveillance and patient care in a major effort to reduce dengue-related deaths (*34*). The aim of this policy is to rapidly identify patients at risk of developing DHF and to initiate prompt, adequate treatment (e.g., intravenous fluid infusion) to prevent DSS. As result of this early and effective replacement of plasma loss, hemoconcentration (one of the classification criteria for DHF) has become a less frequently observed event during the course of the disease, and therefore, a proportion of these cases may not fulfill DHF criteria. Different applications or interpretations of this case definition by countries may limit the ability to make valid comparisons, particularly in Latin America compared to Southeast Asia (*23**,**33*).

The current epidemiologic trend underscores the importance of dengue/DHF and the need for long-term improvements in disease control and surveillance in Brazil. We have described the changing patterns and epidemiologic profile of dengue/DHF during the last 20 years in one of the most severely affected countries in the Americas, highlighting the recent increase in disease severity.
